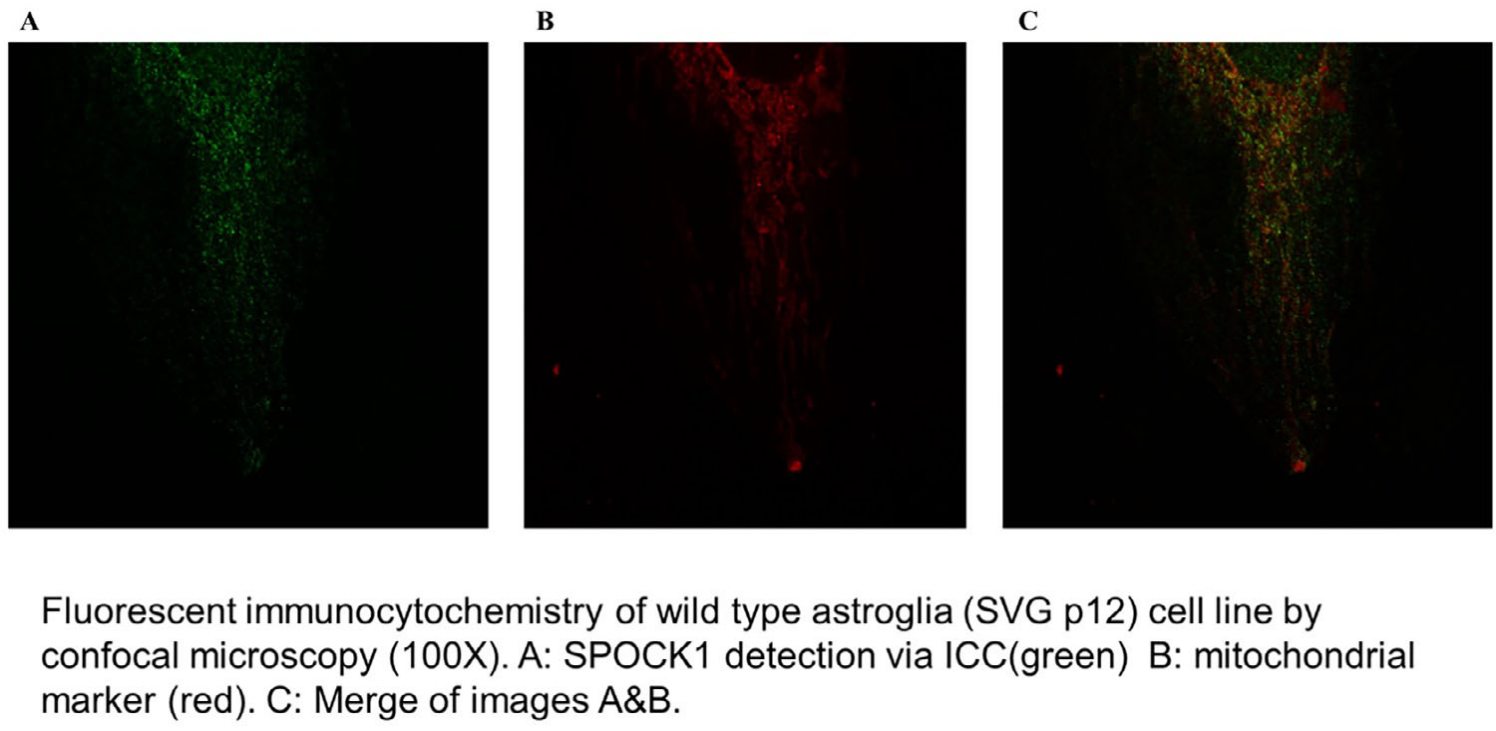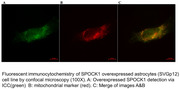# Investigation of Alzheimer's Disease‐Associated effect of SPOCK1 Protein on Apoptosis in Nerve Cells

**DOI:** 10.1002/alz70855_105818

**Published:** 2025-12-23

**Authors:** Emilia Qomi Ekenel, Pardis Safernezhad, Deniz Cansen Kahraman, Çağdaş Devrim Son, Yeşim Aydın Son

**Affiliations:** ^1^ Middle East Technical University, Ankara, Turkey

## Abstract

**Background:**

SPOCK1, a member of the SPARC family, is highly expressed in breast, lung, prostate, and liver cancer. Various studies have demonstrated that SPARC family proteins play a crucial role in the development and diseases of the central nervous system (CNS). However, the role of SPOCK1, a member of the SPARC family, in CNS diseases—particularly Alzheimer's disease—remains unknown. Previously, we have reported an association between SPOCK1 variants and LOAD in a genome‐wide association studies (GWAS) meta‐analysis. Based on our observation and the recent literature on SPOCK1, we explored the role of SPOCK1 in different neuronal cells to understand its possible function in Alzheimer's pathogenesis.

**Methods:**

Human astroglia (SVGp12), microglial (HMC3), and neuroblastoma (SH‐SY5Y) cell lines expressing fluorescently tagged SPOCK1 and un‐tagged SPOCK1 were used to investigate the expression and localization of SPOCK1 via immunocytochemistry (ICC) and confocal image analysis. It is well known that Alzheimer's disease is associated with death mechanisms in nerve cells. To investigate the molecular basis of Alzheimer's disease based on the possible unpredicted interactions and roles of SPOCK1 protein in mitochondrial cell death, SPOCK1 gene silencing and over‐expression effects on apoptosis were detected via TUNEL assay in human astroglia (SVGp12), microglial (HMC3), and neuroblastoma (SH‐SY5Y) cell lines.

**Results:**

Our results based on immunocytochemistry (ICC) and confocal image analysis with various organelle markers showed for the first time that SPOCK1 is localized at the mitochondria of all the microglial, neuroblastoma, and astroglia cells. Moreover, apoptosis was detected in SPOCK1 over‐expressed astrocyte cells. However in SPOCK1 over‐expressing neuroblastoma cells, proliferaiton is observed, and when SPOCK1 was silenced, apoptotic cells were decreased. In microglial cells, no significant change was observed when SPOCK1 was over‐expressed or silenced.

**Conclusions:**

For the first time we have shown localization of SPOCK1 in mitochondria in astroglia, microglial, and neuroblastoma cell lines, and studied SPOCK1's effect on cell death in these cell lines. The SPOCK1 overexpression in astrocytes increased apoptosis, highlighting its potential role in cell death. Overall our findings suggest a potantial role for SPOCK1 in Alzheimer's etiology and introducing it as a novel molecule in neurodegenerative diseases.